# Effectiveness of an Electronic Automated Antibiotic Time Out Alert in the Setting of Gram-Negative Bacteremia

**DOI:** 10.3390/antibiotics10091078

**Published:** 2021-09-06

**Authors:** Sana M. Mohayya, Navaneeth Narayanan, Daniel Cimilluca, Alexander Malanowski, Parth Vaidya, Tanaya Bhowmick

**Affiliations:** 1 Department of Pharmacy, Robert Wood Johnson University Hospital, New Brunswick, NJ 08901, USA; navan12@pharmacy.rutgers.edu; 2 Department of Pharmacy Practice and Administration, Ernest Mario School of Pharmacy, Rutgers University, Piscataway, NJ 08854, USA; parthv18@gmail.com; 3 Department of Medicine, Division of Allergy, Immunology, Infectious Diseases, Rutgers Robert Wood Johnson Medical School, New Brunswick, NJ 08903, USA; ajm421@rwjms.rutgers.edu (A.M.); bhowmita@rwjms.rutgers.edu (T.B.); 4 Department of Pathology and Laboratory Medicine, Robert Wood Johnson University Hospital, New Brunswick, NJ 08901, USA; dc1251@rwjms.rutgers.edu

**Keywords:** antimicrobial stewardship, antibiotics, antimicrobial prescribing, behavior change

## Abstract

To minimize complications associated with over-utilization of antibiotics, many antimicrobial stewardship programs have incorporated an antibiotic time out (ATO); however, limited data are available to support its effectiveness. This was a single-center retrospective cohort study assessing the impact of the automated electronic ATO in the setting of Gram-negative bacteremia. The primary outcome was the proportion of patients who received a modification of therapy within 24 h of final culture results. Secondary outcomes included modification at any point in therapy, time to modification of therapy, time to de-escalation, and days of therapy of broad-spectrum antibiotics. There was a total of 222 patients who met inclusion criteria, 97 patients pre-ATO and 125 patients post-ATO. The primary outcome of modification of therapy within 24 h of final culture results was not significantly different (24% vs. 30%, *p* = 0.33). The secondary outcome of modification of therapy at any point in therapy was not significantly different between the two groups (65% vs. 67%, *p* = 0.73). All other secondary outcomes were not significantly different. The ATO alert was not associated with a higher rate of antibiotic modification within 24 h of culture results in patients with GNB. Further efforts are needed to optimize the ATO strategy and antibiotic prescribing practices.

## 1. Introduction

While broad-spectrum antibiotics may be effective to treat a variety of infections, their over-utilization may lead to superinfections and contribute to the development of antimicrobial resistance. In an effort to minimize these complications, antimicrobial stewardship programs have been developed with the goal of optimizing antibiotic therapy by using the most targeted regimen whenever possible. Stewardship programs include a variety of effective strategies to optimize antimicrobial therapy, including an antibiotic time out (ATO). An ATO is a strategy endorsed by the Centers for Disease Control and Prevention (CDC) and the Joint Commission (TJC), which encourages a re-evaluation of antibiotic therapy at a pre-specified time point during empiric treatment after clinical results have been reported [1,2]. Despite the increasing adoption of an ATO, the optimal strategy and timing have not yet been established.

Previous studies [3,4,5] have assessed the efficacy of an ATO strategy involving prospective audit and feedback (PAF) in which an infectious diseases (ID) physician or pharmacist reviews individual patient cases and intervenes when a change in therapy is deemed appropriate. These studies suggested that this type of intervention may be effective as an adjunct to other stewardship interventions, but the impact of a self-driven ATO was uncertain. A more recent study [6] evaluated the concept of self-stewardship, which prompts the primary care provider to re-evaluate therapy when more data was available and found that providers were more likely to de-escalate antibiotics. The results of these studies have been inconsistent as some have shown utility while others have not [3,4,5,6] and the optimal method of delivery of this alert has not yet been demonstrated. This study aimed to address these inconsistencies by analyzing an automated ATO in patients who have a true infection and who received at least 72 h of antibiotic treatment. We chose to focus on Gram-negative bloodstream infections (GNB) because it is classified as a definitive disease state without concern for the possibility of colonization or misclassification. GNB is a high yield stewardship research opportunity for modification of therapy, as it is common practice to de-escalate or escalate and, therefore, may have a better yield in terms of clinical impact [7].

ATOs have been adopted broadly as an antimicrobial stewardship tool, despite limited evidence supporting their effectiveness. This study was designed to analyze an automated tool built into the electronic medical record (EMR). The objective of the study was to assess the impact of an automated EMR-integrated ATO on the proportion of antibiotic modification for patients with Gram-negative bacteremia (GNB).

## 2. Results

**Figure 1:**
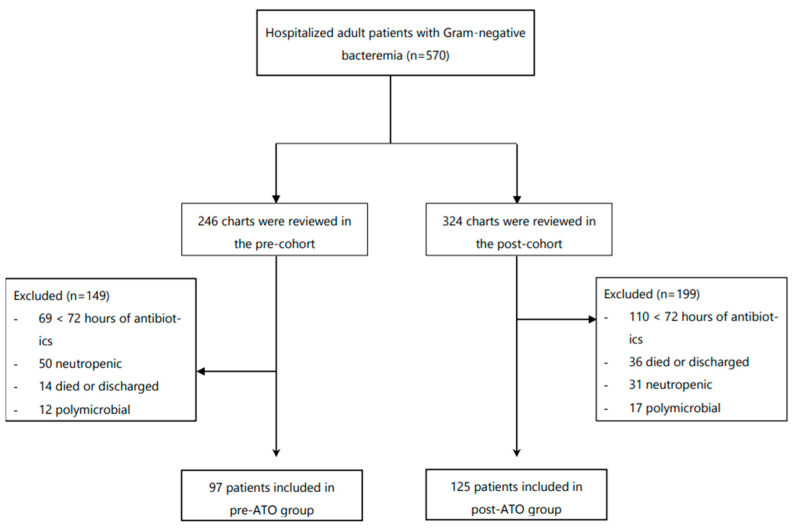
Study flowchart.

Of the 570 adult inpatients with GNB between September 2016 and December 2018, 222 patients were eligible to be included in the study (Figure 1). A total of 97 patients were included in the pre-ATO cohort and 125 patients in post-ATO cohort. Patient demographics and other covariates were assessed (Table 1). Overall, there were no statistically significant differences in baseline characteristics between the two cohorts. The predominant source of bacteremia was urinary (95/222 (43%)) and the most common isolated taxon was *E. coli* (127/222 (57%)). All other encounter characteristics, including Pitt bacteremia score and ICU admission, were similar between the two groups. All positive cultures resulted by time of decision making.

The primary outcome of modification of therapy within 24 h of final culture results was not significantly different for patients in the pre-ATO and post-ATO groups (24% vs. 30% (*p* = 0.33)), as shown in Table 2. The secondary outcome of modification of therapy at any point in therapy after final culture results was not significantly different between the two groups (65% vs. 67%, *p* = 0.73). Of the 147 patients who received a modification of therapy, the mean time to modification from time of final culture results was not significantly different between the two cohorts (27.2 h vs. 25.4 h, *p* = 0.09). All other secondary outcomes were not significantly different between study groups including days of broad-spectrum antibiotic therapy.

An exploratory subgroup analysis of patients who did not achieve the primary outcome was performed to assess for differences among these patients (Table 3). A total of 75 patients were included (34 patients from the pre-cohort and 41 patients from the post-cohort). There were no significant differences in the characteristics, including ICU admission (29% vs. 29%, *p* = 0.99) and vasopressor use (21% vs. 17%, *p* = 0.93) during therapy. Resistance patterns were also similar (*p* = 0.57). Within this subgroup, we also assessed if the infectious diseases team was consulted (59% vs. 66%, *p* = 0.70), as well as if the consult occurred prior to culture results (44% vs. 39%, *p* = 0.42).

A separate analysis of the ATO data was performed for the intervention cohort (supplementary material: Table S1). Among the 125 patients included in the intervention cohort, 109 patients (87.2%) triggered the automated ATO within the EMR. Of those, 36 patients (28.8%) achieved the primary outcome of modification within 24 h of final culture results. A total of 88 patients (70.4%) had a modification of therapy at any point in the hospitalization.

## 3. Discussion

In this observational study evaluating the effect of an automated ATO alert in patients with GNB, we did not find a significant difference in the primary outcome. While more patients in the post-implementation cohort had a modification of antibiotic therapy within 24 h of final culture results, this difference was not statistically significant. In addition, the secondary outcome of modification at any point in therapy was not statistically significant between the two cohorts. The time to modification was shorter in the post-cohort, however this difference was not significant. The other secondary outcomes of 14-day in-hospital mortality, days of therapy, and incidence of *C. difficile* infections were also not found to be significant.

Our results may be comparable to Gruber et al. [3] who implemented an electronic antimicrobial dashboard that required physicians to complete an electronic form and a documented note within the EMR. The authors measured discontinuations of vancomycin and piperacillin-tazobactam, two of the most commonly used broad-spectrum agents. The authors found a higher rate of discontinuation of vancomycin, but, similar to our study, also found no difference for piperacillin-tazobactam. Our study included other broad-spectrum antibiotics and focused primarily on GNB, but the results were comparable.

Similar to other studies, we found that an automated ATO alert may not be as effective as the sole stewardship intervention. A passive automated alert in addition to an active intervention may be most effective. Thom et al. [5] evaluated the effect of a provider-driven paper ATO tool that consisted of a structured conversation during clinical rounds, prompted by the provider without direction from the stewardship team, and subsequent completion of a form which included current antibiotic data. The authors found a significant difference in the proportion of modification or discontinuation of antibiotics, which was analyzed as a secondary outcome. The contrasting results may indicate that an active provider-led intervention may be more effective than a more passive, automated alert as we analyzed in our study. Similarly, other studies which have assessed an active in-person ATO have reported comparable results [3,8].

Wolfe et al. [6] conducted a retrospective study evaluating all positive blood cultures including both Gram-positive and Gram-negative organisms and found that there was a significant increase in antibiotic de-escalation after the implementation of the automated ATO. The results of our study may differ largely be due to the patient population included, as Wolfe et al. included patients with all types of infections. Our study only included Gram-negative infections, which are generally more severe infections and may be a reason for prescribers to be less inclined to de-escalate antibiotics.

The timing of the alert may have been a factor affecting our results. The ATO was designed to trigger after 72 h of antibiotic initiation to allow ample time for final cultures and sensitivities to be reported. At this point, teams would be able to make an informed and confident decision to de-escalate, escalate, or appropriately continue antibiotics. A study conducted by Van Schooneveld et al. [9] evaluated an ATO led by a pharmacist which was performed 72 h after antibiotic initiation and after ≤5 days of initiation. The authors did not find a significant difference in antibiotic use, which suggests that timing of the intervention may not be a significant factor.

Paulson et al. [10] also evaluated a pharmacist-led ATO intervention at 48 h after antibiotic initiation and evaluated antibiotic use after 72 h. The authors evaluated antibiotic use by documentation of an antibiotic plan in the EMR, which was significantly improved in the ATO group. This suggests that the 72 h timepoint may be appropriate. This study also further suggests our earlier point that an active intervention as part of an ATO may be more effective.

We hypothesized that the implementation of a physician-driven ATO would change physician practice in favor of antimicrobial stewardship measures. However, this was not observed. We speculate that this may be due to alert fatigue, as prescribers at our institution also encounter other alerts during order entry. In addition, despite widespread education of providers prior to and during implementation, further interventions may have been required, especially amongst newer prescribers.

Limitations of this study include the retrospective design of the study, which would make it difficult to control for confounding factors. Another limitation is that the ATO was designed to alert if an order was active for 72 h. Therefore, it would not account for initial one-time orders, which was true for most patients who initiated therapy in the emergency department. Our study did include one-time orders when calculating the duration of an antibiotic, and therefore the timing may not have correlated accurately with the timing of the alert for the provider in real-time. Moreover, the automated ATO allowed physicians to defer the alert, which may also have had an effect on the time when antibiotic modification was performed. However, the alert was intended to act as a reminder so even if action was not taken at that time, it may have prompted a discussion or reassessment which could lead to discontinuation at a later point. Furthermore, the EMR triggered an alert if one particular antibiotic order was active for 72 h so it would be less likely to alert for patients whose antibiotics had been reordered. For example, a dose modification resulting in the discontinuation and re-entry of the antibiotic would postpone the alert. In addition, the ATO was designed to alert only during daytime hours, so any patient eligible for the alert during evening or overnight hours may not have received the alert. It is also important to note that not all in the post-intervention cohort was eligible to receive the ATO. The authors designed the study to analyze real-world impact of the alert, rather than only eligible patients. Moreover, as mentioned earlier, the ATO implemented at our institution was a passive intervention. To build upon our results, it may be beneficial to incorporate other active stewardship tools. For example, our institution recently initiated an antimicrobial stewardship response team. This team, consisting of infectious diseases physicians, faculty members, and pharmacists, contacts providers when culture results are reported so they may provide guidance on treatment. Further studies may look into the effect of this program, or a similar intervention, in addition to an ATO. Another limitation was that there is not a well-established sample size in the literature to calculate sample size. Also, the sample is constrained by the implementation study period so our study was a convenience sampling. It is possible that our study is underpowered but because many of the referred studies also had similar results, this may not be the case.

There are also several strengths of our study. This is the first study assessing the effect of an ATO in a focused population of GNB. By including only GNB, we mitigate the risk of confounding factors due to the type of infection and inappropriate discontinuation of therapy. Another strength was that all patient-related factors, other than the intervention, were similar between the two groups, indicating minimal seasonal or secular trends that could introduce confounding factors.

An effective ATO would optimize antimicrobial usage by encouraging providers to perform timely modification of therapy. This may lead to a reduction in the duration of broad-spectrum antibiotics and, as a result, decrease the possible induction of drug resistance and other associated adverse effects. In addition, with increasing antibiotic resistance, an ATO would alert providers to escalate therapy in the case of bacteria that are resistant to the empiric antibiotic chosen. Due to its considerable potential, the ATO is being increasingly adopted across a variety of platforms and methods. This study was designed to evaluate a passive provider-driven EMR delivered ATO in patients with GNB.

While this data may not support the use of an ATO as the sole stewardship intervention, it may provide further insight into the future of antimicrobial stewardship. As clinicians continue to discover the optimal stewardship strategy, the role of the ATO may be become more pronounced. Other forms of stewardship tools, such as active in-person interventions, may be an important component of an ATO. Further studies investigating the role of an automated time out in combination with other active interventions are needed.

## 4. Materials and Methods

### 4.1. Study Design

This was a single-center, retrospective, observational, before-and-after cohort study conducted at a 625-bed academic medical center (Robert Wood Johnson University Hospital, New Brunswick, NJ, USA). The data was collected via EMR chart reviews of inpatient encounters from January 2018 to December 2018 (intervention cohort) and September 2016 to September 2017 (historical cohort). The sampling was conducted using convenience sampling of first episode per patient during the specified study period.

The EMR used at the institution is Sunrise Clinical Manager (Allscripts, Chicago, IL, USA). The study was approved by the local Institutional Review Board at Rutgers University in New Brunswick, NJ, USA.

### 4.2. Participants

We included hospitalized adults (≥18 years) with GNB, as indicated by a positive blood culture with a Gram-negative pathogen, who received at least 72 h of empiric antibiotics at any point in hospitalization. Only Gram-negative isolates were included as these were likely indicative of true infections. Patients who received less than 72 h of empiric antibiotics would not have triggered the alert. Patients were excluded if they had neutropenia, or absolute neutrophil count (ANC) below 500 cells/m^3^ at time of antibiotic initiation and during empiric therapy. These neutropenic patients would be more likely to continue broad-spectrum antibiotics for longer duration, independent of clinical response or culture results. Patients were also excluded if antibiotics were initiated prior to the patient’s admission on the basis of the outside hospital’s culture results since therapy would already be targeted to recovered pathogen, or if more than one pathogen was identified in the set of blood cultures as other culture results may impact the provider’s decision to continue or modify antimicrobial therapy.

### 4.3. Routine Work-Up and Reporting from the Microbiology Laboratory

The microbiology laboratory is located on-site at Robert Wood Johnson University Hospital. All positive blood culture Gram stain results are called to the nurse caring for the patient who then informs the patient’s physician. Final culture results with identification of organism and susceptibility to antimicrobials are available in the EMR. During both the historical and intervention cohort time periods, there were no changes to laboratory processes or reporting of positive blood cultures as positive. No rapid diagnostic testing strategies were employed for identification of Gram-negative organisms from blood culture isolates. In addition, the antimicrobial stewardship program was not directly involved with routine review, reporting, or guidance of Gram-negative blood culture isolates during the entire study period.

### 4.4. Intervention

The ATO was an automated dashboard built into the EMR displayed to ordering providers. This was implemented in October 2017. The alert was triggered after a patient received at least 72 h of any antibiotic and after a provider entered any new order for that patient in an effort to avoid interruption of workflow. The dashboard displayed the patient’s relevant data, including culture results (if any), temperature curve, laboratory trends, and current antibiotics (supplementary material: Figure S1). The user had the option to either continue or discontinue each antibiotic within that screen. The user was also given the option to defer, in the event that the user cannot make a decision on the basis of the results at that time. Deferring the alert would cause the alert to trigger again after 12 h for that individual provider. In addition, the alert did not fire overnight when the on-call providers were less likely to make decisions on patients for whom they were not the primary care provider. The ATO was designed to alert providers when there may be a potential to optimize antimicrobial therapy in a timely and convenient manner. Educational efforts were provided to all ordering providers prior to the implementation via in-person sessions and electronic distribution through the medical staff newsletter.

### 4.5. Outcomes

The primary outcome was the proportion of patients who received a modification of therapy for GNB within 24 h of final culture results. Modification of therapy was defined as either a de-escalation to a targeted agent or escalation of therapy to broader coverage based on susceptibilities of blood cultures. De-escalation was defined as a change in antibiotic from a broad-spectrum agent to a more targeted agent on the basis of the results of the culture. Escalation was a change in antibiotic to an agent with a broader spectrum on the basis of the results of the culture. Secondary outcomes included modification at any point in therapy and time to modification among those patients. All outcomes measuring time were measured from the time of final culture susceptibilities reported (“time zero”). Other outcomes included 14-day in-hospital mortality, *C. difficile infection* incidence, and days of therapy of empiric antibiotics.

### 4.6. Statistical Analysis

Continuous data were reported as means with standard deviations or medians with interquartile ranges (IQR), as appropriate. All categorical data were reported as percentages. Continuous data were analyzed with using Student’s *t*-test or Wilcoxon rank sum test for nonparametric distribution. Categorical data were analyzed using the chi-squared test or Fisher’s exact test, as appropriate. The significance level was determined as a *p*-value of <0.05 (two-sided). A subgroup analysis of baseline characteristics was performed for patients who did not achieve the primary outcome. Data were analyzed using R software (version 1.0.136).

## 5. Conclusions

In conclusion, the results of our study indicate that a provider-driven automated ATO did not have a significant effect on modifying antibiotic therapy following culture results. However, future studies are required to determine the optimal timing of the alert, especially in the setting of rapid diagnostics. By optimizing the ATO, we can improve antimicrobial stewardship on a global scale and ultimately lessen the risk of antimicrobial resistance.

## Figures and Tables

**Table 1:** Baseline demographics and clinical characteristics of patients included in the study.

Characteristic	Pre-Intervention (*n* = 97)	Post-Intervention (*n* = 125)	*p*-Value
Patient demographics			
Age, y, mean (SD)	66 (50–82)	69 (55–83)	0.62
Female sex	43 (44)	63 (50)	0.42
CKD	25 (26)	40 (32)	0.37
Encounter characteristics			
Pitt Bacteremia Score, mean (SD)	2 (0–5)	2.2 (0–5)	0.76
ICU admission while on antibiotic	38 (39)	44 (35)	0.58
Vasopressor use	20 (21)	31 (25)	0.52
Isolated taxon			0.63
*Escherichia coli*	55 (57)	72 (58)	
*Klebsiella* spp.	19 (20)	28 (22)	
*Proteus mirabilis*	5 (5)	10 (8)	
*Enterobacter* spp.	6 (6)	9 (7)	
*Serratia marcescens*	5 (5)	4 (3)	
*Pseudomonas aeruginosa*	2 (2)	0 (0)	
*Stenotrophomonas maltophilia*	2 (2)	1 (0.8)	
*Acinetobacter baumannii*	1 (1)	1 (0.8)	
*Citrobacter freundii*	2 (2)	0 (0)	
Antibiotic resistance ^a^			0.25
Carbapenem-resistant	6 (6)	2 (2)	
Ceftriaxone-resistant	18 (19)	23 (18)	
Fluoroquinolone-resistant	28 (29)	29 (23)	
Allergies ^b^	22 (23)	32 (26)	0.64
Beta-lactam allergy	18 (19)	20 (16)	
Antibiotic allergy in addition to beta-lactam	6 (6)	5 (4)	
Other antibiotic allergy(non-beta-lactam)	4 (4)	12 (10)	
Anaphylaxis reported	5 (5)	5 (4)	
Likely source of bacteremia			0.65
Urinary only	38 (39)	57 (46)	
Abdominal only	25 (26)	27 (22)	
Respiratory only	8 (8)	6 (5)	
Other site	12 (12)	16 (13)	
Multisite	7 (7)	13 (10)	
Unknown	5 (5)	2 (2)	
Skin and soft tissue only	1 (1)	3 (2)	
Other positive culture ^c^			
Same pathogen as index blood culture	26 (27)	48 (38)	0.70
Same sensitivity profile as index blood culture ^d^	22 (85)	40 (83)	
Focus of infection ^e^	29 (30)	45 (36)	0.42
Removed	20 (69)	36 (80)	
Not removed	9 (31)	9 (20)	
Antibiotic at time of final culture results			
Piperacillin-tazobactam	61 (63)	75 (60)	0.82
Ceftriaxone	10 (10)	16 (13)	
Meropenem	9 (9)	15 (12)	
Other ^f^	17 (18)	19 (15)	
Modified antibiotic ^g^			0.93
Ceftriaxone	18 (28)	25 (30)	
Levofloxacin	12 (19)	18 (21)	
Cefazolin	8 (13)	10 (12)	
Meropenem	8 (13)	7 (8)	
Other ^h^	17 (27)	24 (29)	
Primary hospital service at time of culture results			0.62
Internal medicine	47 (48)	77 (62)	
Pulmonary	22 (23)	26 (21)	
Surgical	13 (13)	14 (11)	
Cardiovascular	4 (4)	4 (3)	
Other ^i^	11 (11)	4 (3)	
Discharge status			0.38
Home	52 (54)	56 (45)	
Institution ^j^	32 (33)	52 (42)	
Death ^k^	13 (13)	17 (14)	

Data are presented as no. (%), unless otherwise indicated. Abbreviations: CKD, chronic kidney disease; ICU, intensive care unit; ID, infectious disease. ^a^ Category of resistance is mutually exclusive and only highest level of resistance was recorded. Intermediate resistance patterns were considered resistant. ^b^ Of the patients who reported a beta-lactam allergy, 33% of the pre-cohort and 4% of the post-cohort had additional antibiotic allergies. Anaphylaxis included patients with allergy to any antibiotic with documentation of serious anaphylactic reactions, including symptoms of shortness of breath and swelling. ^c^ A positive culture from another source (i.e., urine, tissue, body fluid) that had final results prior to or within 24 h of final blood culture results. ^d^ Proportion of other cultures with same sensitivities are reported on the basis of the frequencies of cultures with same pathogen as blood culture. ^e^ Removed focus of infection includes patients who had a source of infection that may require removal to achieve source control. The percentages reported are based on the total number of patients who had a removable source of infection. ^f^ Other antibiotics include aztreonam, levofloxacin, ertapenem, ceftazidime, cefazolin, cefepime, ampicillin-sulbactam, ciprofloxacin, ceftazidime-avibactam, and ceftolozane-tazobactam. ^g^ If antibiotic was modified. Percentages are calculated based on the total number of patients who modified therapy. ^h^ Other antibiotics after modification included amoxicillin, amoxicillin-clavulanate, ampicillin-sulbactam, sulfamethoxazole-trimethoprim, ceftazidime, ceftazidime-avibactam, ceftolozane-tazobactam, cephalexin, ciprofloxacin, and ertapenem. In cases when escalation of therapy was indicated, piperacillin-tazobactam and meropenem were initiated. ^i^ Other services include hematology/oncology, gynecology/oncology, neurology, heart transplant, infectious disease, and nephrology. ^j^ Institution is defined as discharged to another institution, including another hospital, rehabilitation center, long-term care facility, or skilled nursing facility. ^k^ Death includes patients who were discharged to hospice.

**Table 2:** Primary and secondary outcomes.

	Pre-Intervention (*n* = 97)	Post-Intervention (*n* = 125)	*p*-Value
Primary outcome			
Modification within 24 h of culture results ^a^	23 (24)	37 (30)	0.33
Secondary outcomes			
Modification at any point in therapy	63 (65)	84 (67)	0.73
Time to modification (hours)–median (±IQR) ^b^	27.2 (21–52)	25.4 (10–48)	0.09
14-day in-hospital mortality	8 (8)	12 (10)	0.73
DOT ^c^ of broad-spectrum antibiotic–median (±IQR)	6 (2–10)	5 (2–8)	0.08
*C. difficile* infection	2 (2)	9 (7)	0.08

Data are presented as no. (%), unless otherwise indicated. Abbreviations: AKI, acute kidney injury; DOT, days of therapy. ^a^ Therapy modification is defined as the action of de-escalating to targeted regimen or escalating to broader coverage based on culture results. One patient in the pre-cohort was classified as a discontinuation of antibiotics due to allergic reaction to the empiric therapy and subsequent modification of therapy to an inactive agent. ^b^ Time to modification among patients who modified at any point in therapy. ^c^ Days of therapy was calculated from day of therapy initiation through day of discontinuation. Broad-spectrum antibiotic includes piperacillin-tazobactam, ceftriaxone, meropenem, aztreonam, levofloxacin, ertapenem, ceftazidime, cefazolin, cefepime, ciprofloxacin, ceftazidime-avibactam, and ceftolozane-tazobactam.

**Table 3:** Subgroup analysis of patients who did not achieve primary outcome.

Characteristics	Pre-Intervention (*n* = 34)	Post-Intervention (*n* = 41)	*p*-Value
ICU admission while on antibiotic	10 (29)	12 (29)	0.82
Vasopressor use	7 (21)	7 (17)	0.99
Antibiotic resistance			0.57
Carbapenem resistant	1 (3)	1 (2)	
Ceftriaxone resistant	5 (15)	11 (27)	
Fluoroquinolone resistant	7 (21)	3 (7)	
ID consult	20 (59)	27 (66)	0.70
ID consult before culture results	15 (44)	16 (39)	0.42

## Data Availability

The datasets generated during and/or analyzed during the current study are available from the corresponding author upon reasonable request.

## Supplementary Materials

The following are available online at https://www.mdpi.com/article/10.3390/antibiotics10091078/s1, Table S1: Antibiotic time out data for intervention cohort. Figure S1: Example of time out alert.
