# Supplementary material for: Effectiveness of an Electronic Automated Antibiotic Time Out Alert in the Setting of Gram-Negative Bacteremia
Source: Antibiotics (Basel). 2021 Sep 6;10(9):1078. doi: 10.3390/antibiotics10091078 (PMC8466937; doi:10.3390/antibiotics10091078)
Supplement: Supplementary file 1 [file antibiotics-10-01078-s001.zip › antibiotics-1340856-supplementary.pdf]

Supplementary Materials

Antibiotic Time Out (ATO) Data

Table S1. Antibiotic Time Out Data for Intervention Cohort.

Antibiotic at Time of Alert	Eligible for ATO Alert	Modified within 24 hours of culture results	Modified at any point in therapy (after 24 hours)
**Piperacillin-tazobactam**	65	27	59
**Ceftriaxone**	15	0	5
**Meropenem**	13	5	12
**Levofloxacin**	5	1	3
**Ertapenem**	4	1	1
**Aztreonam**	4	1	4
**Ceftazidime**	1	0	0
**Ampicillin-sulbactam**	1	0	1
**Ceftazidime-avibactam**	1	0	1
**Cefepime**	0	1	1
**Ceftolozane-tazobactam**	0	0	1
**Grand Total**	**109**	**36**	**88**

Example of ATO Alert

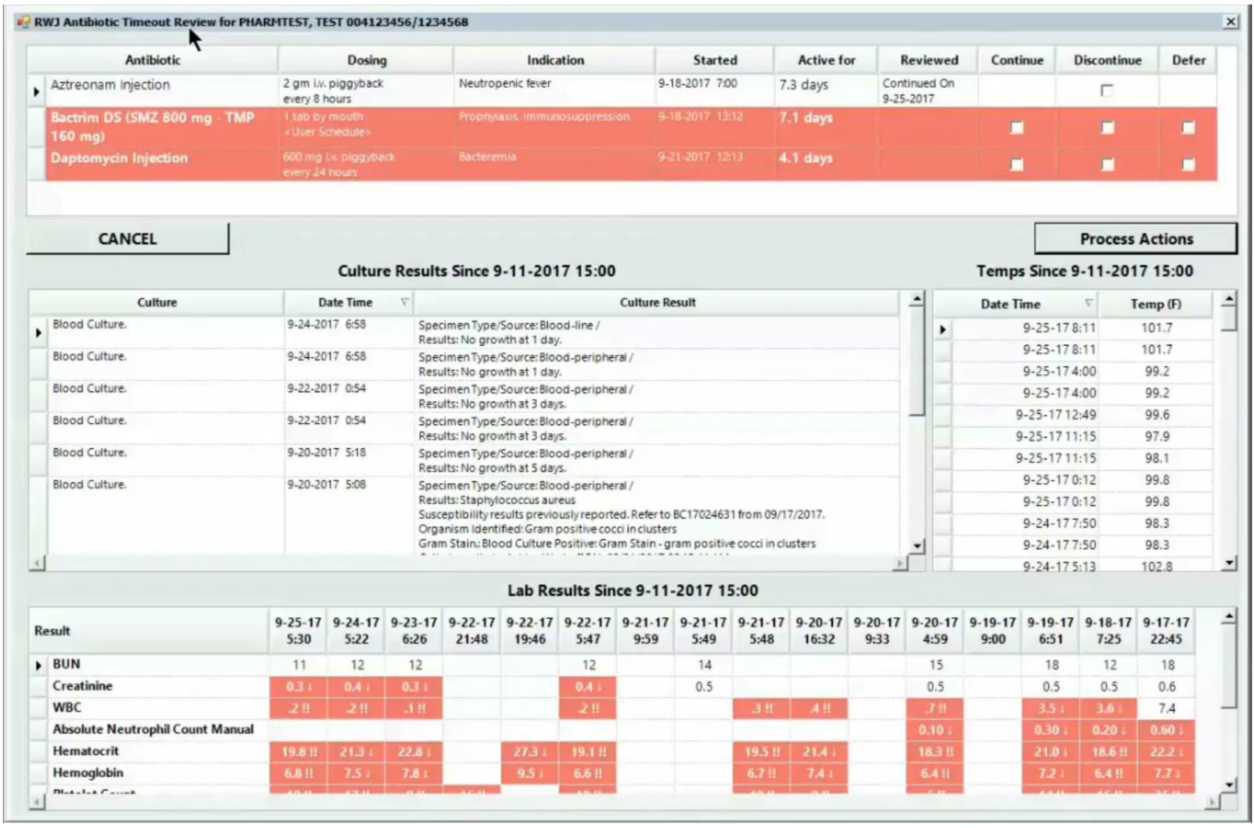

Figure S1. Example of EMR-based ATO alert.
